# Pathogenesis of Cholesterol Gallstones

**DOI:** 10.1155/1991/61741

**Published:** 1991

**Authors:** Steven M. Strasberg, Pierre-Alain Clavien, P. Robert C. Harvey

**Affiliations:** 1 Department of Surgery and the Samuel Lunenfeld Research Institute Mount Sinai Hospital University of Toronto Toronto Canada; 2 Suite 1225 600 University Avenue Toronto M5G 1X5 Canada

## Abstract

Cholesterol gallstone disease is extremely common. Three major stages are recognized for stone formation, namely bile that becomes supersaturated with cholesterol, cholesterol nucleation leading to crystal formation and finally retention of the crystals in the gallbladder resulting in stone formation. Supersaturation is common but nucleation into crystals probably requires protein nucleating factors. Impaired motility of the gallbladder causes crystal retention and is probably very important in stone formation.


*HPB Surgery*, 1991, Vol. 3, pp. 79–102
Reprints available directly from the publisher
Photocopying permitted by license only

© 1991 Harwood Academic Publishers GmbH
Printed in the United Kingdom

### REVIEW

### PATHOGENESIS OF CHOLESTEROL GALLSTONES

STEVEN M. STRASBERG, PIERRE-ALAIN CLAVIEN and P. ROBERT C. HARVEY

*Department of Surgery and the Samuel Lunenfeld Research Institute, Mount Sinai Hospital, University of Toronto, Toronto Canada.*

Cholesterol gallstone disease is extremely common. Three major stages are recognized for stone formation, namely bile that becomes supersaturated with cholesterol, cholesterol nucleation leading to crystal formation and finally retention of the crystals in the gallbladder resulting in stone formation. Supersaturation is common but nucleation into crystals probably requires protein nucleating factors. Impaired motility of the gallbladder causes crystal retention and is probably very important in stone formation.

Cholesterol gallstone disease is one of the most common serious diseases. The incidence of stone disease is highest in the Americas and Europe with incidence reaching 30% in women and 15% in men at age 60 in some populations[1,2]. Mortality rates vary from 2 to 6 per 100,000 per year[2] depending on the associated incidence of gallbladder cancer[2]. For cholesterol gallstones to form, three conditions must be achieved; the bile must become supersaturated with cholesterol, the cholesterol must precipitate as solid cholesterol monohydrate crystals and the crystals must aggregate with other elements of a stone to form the recognizable macroscopic concretion (Figure 1). In this review we will focus on the pathogenesis of cholesterol gallstone disease. The first part of the review will deal with the chemistry of biliary lipids, their secretion into bile and events occurring in bile which result in the formation of cholesterol crystals. The second part will deal with the conditions leading to supersaturation, nucleation and stone formation in human biles.

### *Relevant chemical species in bile. (Cholesterol, Phospholipid, Bile Salts) Figure 2.*

### *Cholesterol*

Cholesterol is the major component of cholesterol gallstones. It is a sterol which is almost totally insoluble in water, the aqueous solubility being about $10^{-8}$mM[3]. Cholesterol accounts for about 95% of all sterols in bile and gallstones[4]. Cholesterol esters do not exist in bile. The importance of the chemical structure of

Address correspondence to: S.M. Strasberg, Suite 1225, 600 University Avenue, Toronto, Canada. M5G 1X5

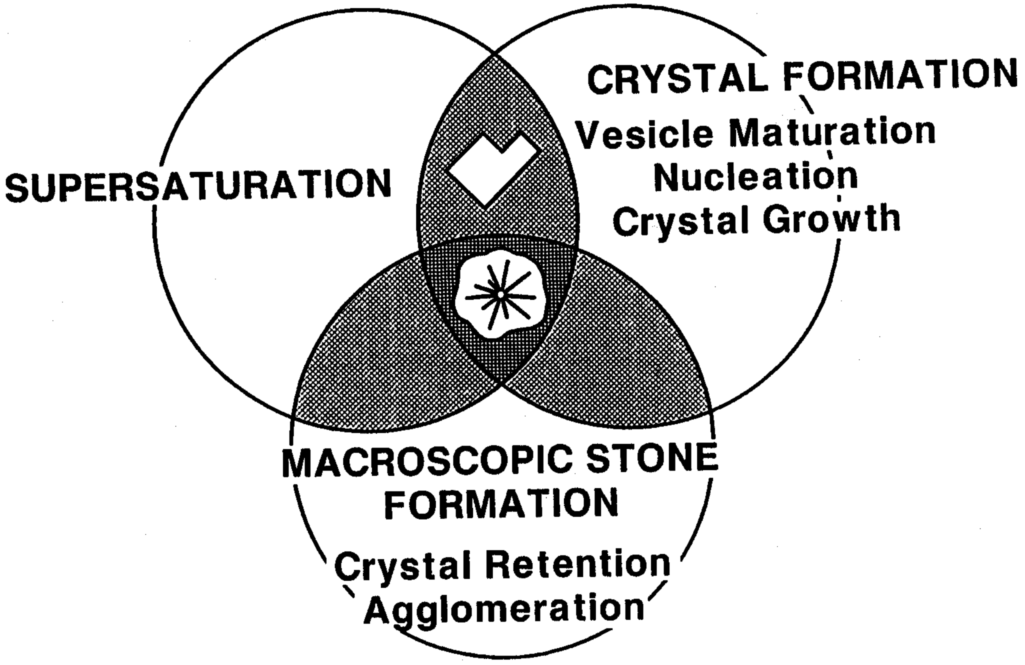

**Figure 1** Venn diagram showing the conditions necessary for cholesterol gallstone formation.

cholesterol is that although biliary secretion is a major route for excretion, the substance is virtually insoluble in water and so carrier systems must be available to transport the cholesterol in bile. These carriers have finite capacities, which if exceeded can lead to the precipitation of cholesterol crystals.

*Phospholipids*

Although there are many different types of hepatic phospholipids, more than 95% of biliary phospholipid is diacylphosphatidylcholine (lecithin). Lecithin consists of a glycerol molecule with two fatty acid chains in the sn–1 and sn–2 positions and a choline group originating from the third glycerol carbon. Lecithins differ in their fatty acid chain composition, with the least hydrophobic hepatic lecithins selected for secretion. The commonest biliary lecithins are 16:0–18:2 and 16:0–18:1, with the pattern of a saturated fatty acid at sn–1 and an unsaturated fatty acid at sn–2 being the common one for most other biliary lecithins also[5].

Some differences in lecithin species have been reported between patients with and without gallstones[6,7], but there is incomplete agreement in the results of these studies and the relevance of the findings is as yet unknown. However, these observations are potentially quite important since phospholipids contain arachidonic acid the precursor substance of prostaglandins, which have important effects on the gallbladder.

Phospholipids also are very sparingly soluble in water as monomers, but swell in water to form a bilayer i.e. sheets two molecules thick, in which the fatty acid

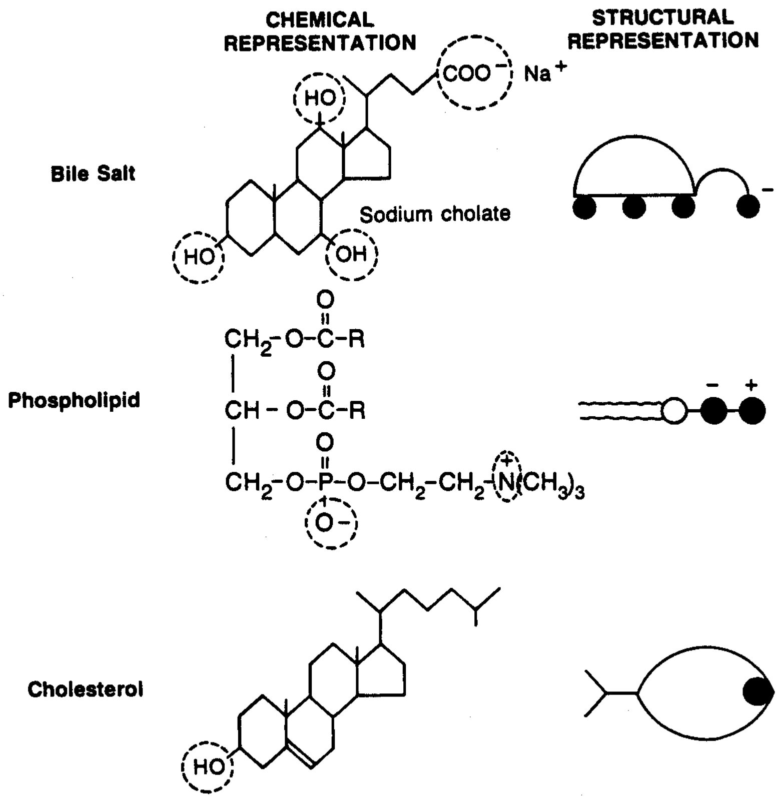

**Figure 2** Chemical species in bile. The three major lipids of interest are shown. Polar groups represented in black in the structural representation have been circled by an interrupted line in the chemical representation.

chains project toward the interior of the sheet and the polar choline groups point outward into the water phase. This is the characteristic positioning of phospholipid in cell walls and also in biliary mixed micelles and vesicles. A vesicle is a phospholipid sheet folded to form a hollow sphere of bilayer, enclosing some of the aqueous medium in the interior. Mixed micelles have a drum like configuration in which the sheet-like bilayer is the skin of the drum and bile salts are the rim. The importance of the bilayer in gallstone formation is that it is in the interior of the bilayer where the fatty acid chains of lecithin provide a highly hydrophobic milieu that increases cholesterol solubility. Precipitation is possible when these sites become overloaded, i.e., supersaturated with cholesterol.

*Bile Salts*

Bile salts are detergent molecules. Their detergent properties are conferred due to the positioning of a large hydrophobic nucleus of the molecule on one side of the molecule and polar hydroxyl and carboxyl groups project on other side. Bile salts are soluble in water but above a very low concentration (about 5mM — the critical micellar concentration or CMC) associate into micelles. The common bile salts in man are cholate (trihydroxy) and chenodeoxycholate (dihydroxy) which are synthesized in the liver (primary bile salts), and deoxycholate (dihydroxy) and lithocholate (monohydroxy) produced from the primary bile salts in the intestine by bacterial dehydroxylation and referred to as secondary bile salts. Bile salts are also conjugated in the liver to taurine or glycine which, by lowering their pKa, allows them to remain ionized in the slightly acidic milieu of the upper small intestine, where otherwise they would be absorbed and not be available for fat digestion. As noted above bile salts have the capacity to solubilize a section of phospholipid bilayer into a mixed micelle, making the drum-like mixed micelle referred to above. Bile salts not only form the rim of the drum but are also inserted into the skin of the drum among the phospholipids, and so this type of micelle is referred to as the mixed-disc micelle[8]. The ability of bile salt to insert into the bilayer is important since this may be necessary for vesicular aggregation which precedes nucleation of cholesterol. (see below)

### *Dynamic and Equilibrium States*

The three lipids and water form an interactive system of molecules. In the cholesterol-lecithin-bile salt-water system cholesterol may be found in at least three phases or physical forms, i.e., micelles, vesicles, and solid cholesterol monohydrate crystals. Cholesterol is also present in extremely low concentration as monomers. The phases which are found are governed by the concentrations of the four components of the system, and the phase(s) present in a particular model bile may be accurately predicted by the equilibrium phase diagram for the system provided the concentrations are known and the system is at equilibrium[9] (Figure 3). However, mixtures of lipids are not necessarily at equilibrium nor do they necessarily move to equilibrium rapidly, and may in fact take several days or weeks to do so. Other components of the system which do not affect the phases present at equilibrium may nonetheless importantly affect the dynamics of the system, slowing or accelerating the time to equilibrium. One may by analogy think of the equilibrium phase diagram as a set of architectural plans; plans depict the static state reached after construction is complete and one may accurately predict from those plans where the windows, doors, etc. will be once the building is complete, i.e. by analogy the static state of equilibrium. However the plans do not permit determination of state of the construction site during any point in the kinetic or dynamic state of active construction, nor do they include information on factors which can accelerate or slow the dynamic process leading to the completion state. This is a key point in understanding several recent advances in this area, particularly as nucleation of cholesterol crystals is a late kinetic event in biological systems.

### *Secretion of Biliary Lipids*

It has been known for many years that bile salts stimulate the secretion of cholesterol and phospholipid into bile[10,13]. This was initially interpreted to indicate that bile salt micelles solubilized liver canalicular membrane phospholipid and cholesterol by detergent action and that the mixed micelles so produced were secreted into bile. The canalicular membrane was the proposed site of bile salt action and it was expected that phospholipid composition of canalicular membrane

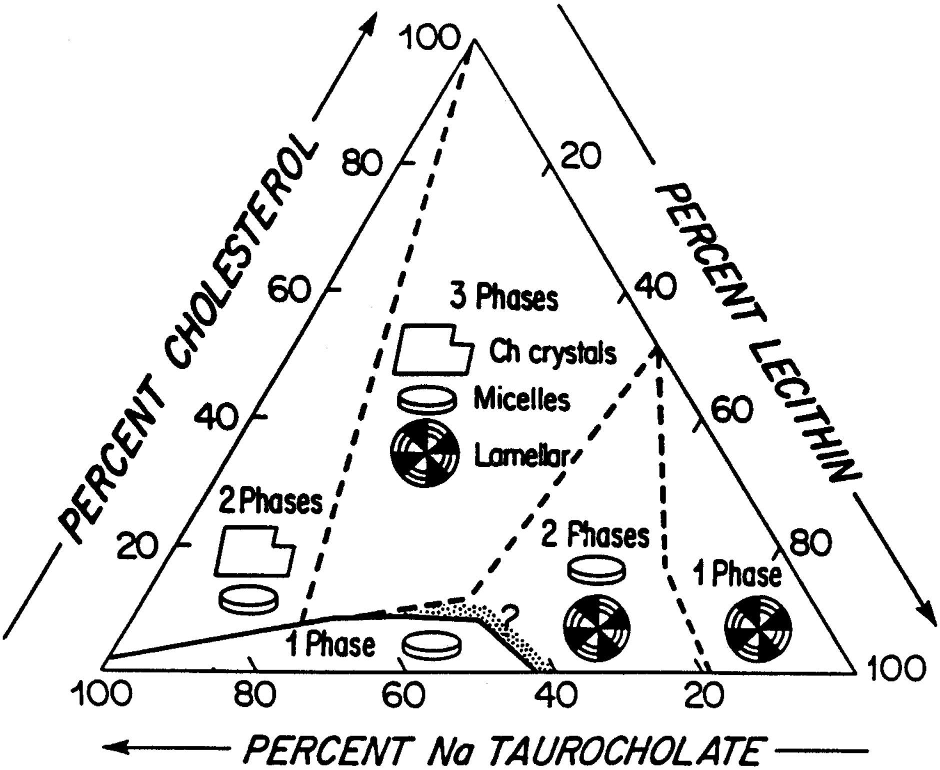

**Figure 3** Equilibrium phase diagram for bile salt-phospholipid cholesterol-water at a concentration of 10% solids 90% water. Note that outside the micellar zone two different phases are possible, cholesterol crystals and vesicles. (From reference 9 by permission)

phospholipids would resemble biliary phospholipids. In fact the composition was found to be quite different[14]; biliary phospholipid is almost entirely phosphatidyl choline, whereas a variety of phospholipid species are present in the canalicular membrane. Nevertheless, it seemed possible that bile salts might act upon phosphatidyl choline (PC) rich domains of the membrane, thus accounting for the particular composition of bile.

These concepts have had to be completely revised recently because of the demonstration of unilamellar vesicle in bile by Carey and coworkers in animals[15] and by Somjen and Gilat in man[16,17]. Unilamellar vesicles have now been demonstrated in the canaliculus itself by Nervi and his colleagues using transmission EM[18]. Cohen *et al.*[19] employing quasielastic light scattering also provide evidence for vesicular secretion of biliary lipids. It is highly likely that biliary phospholipid and cholesterol are co-secreted as vesicles and that the site of origin of the lipid is the smooth endoplasmic reticulum. Biliary phospholipid appears to originate from a rapidly turning over pool at that site[20], whereas most biliary cholesterol i.e., about 80% is preformed and only 20% is newly synthesized[21]. Biliary vesicles move to the canalicular pole of the liver cell probably through the Golgi apparatus and fuse with the canalicular membrane before budding off into the canaliculus[22]. These processes may be inhibited by colchicine, indicating that the cellular microtubular system is important in vesicular movement from the SER to the canaliculus[23]. Bile salts could effect these processes at the SER where they have binding sites[24], at the Golgi or from the canalicular side of the lumen. The latter possibility gains support from the observation that bile salt concentrations in bile are elevated prior to the increase in cholesterol or phospholipid concentrations[25] and the observation that while inorganic anion transport uncouples the effect of bile salts on cholesterol secretion[26], this does not occur in rat strains incapable of anion excretion into the canaliculus, despite the presence of high concentrations of anion in the liver cell[27].

The process of biliary cholesterol secretion must be considered in the context of total body cholesterol metabolism. Bile salts may be thought of as a signal to cholesterol traffic to enter the canaliculus. Other signals received by the liver cell will result in routing of cholesterol into lipoproteins for secretion into the plasma[28]. How these signals are processed and how the proportion of cholesterol and phospholipid in vesicles are determined is unknown. The latter is of extreme interest in understanding cholesterol gallstone formation since the ratio of cholesterol to phospholipid in the vesicle determines to a large extent whether or not the bile will be supersaturated.

Bile salts themselves appear to enter the canaliculus by a carrier mediated transport process[29] and presumably form simple micelles in the canalicular lumen.

### *Maturation of Biliary Lipid Carriers in the Biliary Tree*

Biliary lipids begin to interact after secretion, the end stage of these interactions being the equilibrium state. Maturation is the process by which the secreted lipid carriers are altered in the bile, and maturation, nucleation and crystal growth are the processes leading from the secreted state to the equilibrium state. As noted above, the equilibrium state of a particular bile sample is quite predictable at the moment of secretion from the concentration of the three lipids in the sample, however the rate at which the kinetic processes proceed is what determines whether the bile will ever reach equilibrium within the time-scale that it is in the biliary tree. Thus one supersaturated bile may proceed to equilibrium and solid crystal formation, while another may remain in a pre-equilibrium state and never form crystals while in the biliary tree.

As noted in the previous section it seems that all secreted cholesterol and phospholipid are initially in vesicular form and the C:P ratio of these vesicles is less than 1.0 even in supersaturated biles. Vesicles with a C:P ratio less than 1.0 are highly stable in respect to nucleation of cholesterol[30,31]; even vesicles with C:P ratios of 1.5 require several days to nucleate cholesterol crystals[30]. Maturation involves two processes, progressive micellation of vesicular phospholipid and cholesterol and relative enrichment of vesicular cholesterol, i.e., an increase in the C:P ratio. Micellation is the movement of lipid from vesicle to micelle; studies of lipid movement between vesicles and between micelles in model systems indicate several possible mechanisms including transfer of phospholipid and cholesterol monomers through the aqueous phase and transfer during collision of micelles and vesicles[32,33]. There is direct experimental evidence for progressive micellation in human biles; we have shown that the per cent cholesterol in vesicles in gallbladder bile is less than in hepatic bile[34]. The concept that human bile vesicles become relatively enriched in cholesterol during micellation comes from observations on both human[31] and model biles[35]. We subjected human bile vesicles to progressively greater concentrations of bile salt on a column, to simulate the maturation process. As expected this resulted in progressive micellation, but the process was uneven in respect to cholesterol and phospholipid, with preference given to micellation of the phospholipid[31]. Consequently vesicles were enriched in cholesterol, the C:P ratio rose and nucleation from these vesicles was accelerated[31]. The process of maturation is probably accelerated in the gallbladder. There, due to dehydration of bile, the number of bile salt monomers required to satisfy the CMC is reduced and new micelles are produced. If the volume of bile is reduced to 20% of initial volume by water and electrolyte absorption the mass of new simple micelles produced is substantial. Dehydration may also be important by increasing the probability of collisions between lipid carriers. Recently we examined a population of persons with cholesterol crystals but no gallstones. Their gallbladder bile was more concentrated than bile of a control group without crystals even though the CSI of the two groups was identical[36].

The maturation process in unsaturated and supersaturated bile may be summarized as follows. Unsaturated bile is a condition of micellar excess. At the canaliculus all the cholesterol and phospholipid are in vesicular form and the C:P ratio is low. As bile moves down the biliary tree there is progressive micellation and enrichment of residual vesicles in cholesterol. Eventually, since there is micellar excess, all the vesicular lipid is micellarized. It is at this point that equilibrium is reached. If the relative composition of the bile is plotted on the equilibrium phase diagram, it will fall within the micellar zone. The events described above occur in time and only after all reactions are completed is equilibrium reached; only then will the phases present in bile correspond to those in the equilibrium phase diagram. In contrast to the unsaturated state, the supersaturated state is a condition of micellar insufficiency. In this case after maturation of the lipid carriers is completed, vesicles with a high C:P ratio remain after micelles become completely saturated with cholesterol. As this system continues to move toward equilibrium, cholesterol crystals appear, and at equilibrium either two (crystals and micelles) or three phases (crystals, micelles and vesicles with a reduced C:P ratio) will be present. An important corollary is that while maturation is beneficial or at least not harmful, in undersaturated bile, maturation must be seen as detrimental in supersaturated bile, since it leads to the conditions necessary for nucleation of crystals. As reaching the equilibrium state rapidly in supersaturated biles is undesirable, agents accelerating the lipid transfer processes of maturation must be pronucleating, whereas agents retarding them are antinucleating. Furthermore, altering bile salt composition by replacing bile salts with good micelle forming properties with ursodeoxycholate, a poor micelle forming bile salt, may actually be beneficial by virtue of slowing this maturation process.

### *Nucleation of Cholesterol Crystals*

Nucleation of cholesterol crystals occurs from biliary vesicles. We have shown that cholesterol crystals nucleate from human bile vesicles that have been separated from micelles, but nucleation does not occur from the micelles[31]. Morphological studies also support the view that nucleation occurs from the vesicular phase[37].

Nucleation appears to occur from the small unilamellar vesicles of human bile after they aggregate[38] (Figure 4A) or after they aggregate and fuse into large multilamellar vesicles[38]. The latter are much larger than the unilamellar vesicles secreted by hepatocytes and appear as "Maltese cross" figures on polarizing light microscopy (Figure 4B); Unilamellar vesicles require EM to be seen. Vesicular lipid is not uniformly distributed over the surface of the vesicular bilayer; instead cholesterol-rich and cholesterol-poor zones exist[39]. As the vesicle becomes enriched in cholesterol, the proportion of the surface that is cholesterol-rich will increase. It is not known for sure how aggregation of small unilamellar vesicles (20–100nm) or their fusion to multilamellar vesicles of much larger size (1000nm) contribute to nucleation, but it seems likely that the juxtaposition of zones of high concentration of cholesterol in adjacent vesicles or adjacent lamellae of multilamellar vesicles is important. For aggregation of vesicles to occur, the repulsion of the polar phospholipid head groups on the outer leaflet of the vesicle bilayer must be overcome. This may be how pronucleating substances work. Fusion of unilamellar vesicles into multilamellar vesicles is a very complex process in which the outer leaflets of aggregated phospholipid vesicles must retract to expose the fatty acid tails of the inner leaflets to each other. Exactly how this happens is unknown except that fusogens appear to insert themselves into the bilayer and distort the bilayer in a manner promoting these events.

Nucleation is not the end stage of crystal formation. When nucleation occurs there are very few cholesterol molecules involved and the unit crystal is probably only 4 molecules. However, little is known about the factors controlling the growth of unit crystals to crystals observable in the light microscope.

### *Supersaturation and Cholesterol Gallstone Formation*

Supersaturation is the sine qua non of cholesterol gallstone formation, and there is a linear correlation between the reported CSI of various population groups and the incidence of cholesterol gallstones in that population (Figure 5)[40]. Supersaturation of bile with cholesterol is extremely common in adults on Western diets. In a large group of control biles we recently observed the median CSI to be 1.04, i.e. supersaturated[36], indicating that more than one-half of patients having surgery for non-biliary conditions in our population have supersaturated biles. Many other studies have also demonstrated that supersaturated bile is extremely common in our population[41–43].

The cause of supersaturation seems to be different depending upon whether the patient is thin or obese[44]. Thin patients appear to have a small bile salt pool and a reduced bile salt secretion rate relative to the secretion rates of cholesterol and phospholipids, whereas the obese have a normal bile salt pool but increased secretion of cholesterol[44]. Cholesterol secretion into bile is directly proportional to body weight[45] accounting for the effect of obesity on cholesterol saturation in bile. Obesity promotes supersaturation through another mechanism also, in that cholesterol secretion rates are markedly increased during periods of rapid weight loss as body tissues are mobilized[46]. The thin supersaturated patient is thought to develop a small bile salt pool by virtue of an inappropriately low response to the stimulus to bile salt synthesis, i.e. as the pool is lost in the intestine these individuals fail to respond appropriately by increasing bile salt synthesis levels which would restore the pool[44]. Why or how this occurs is unknown. Others have also described a

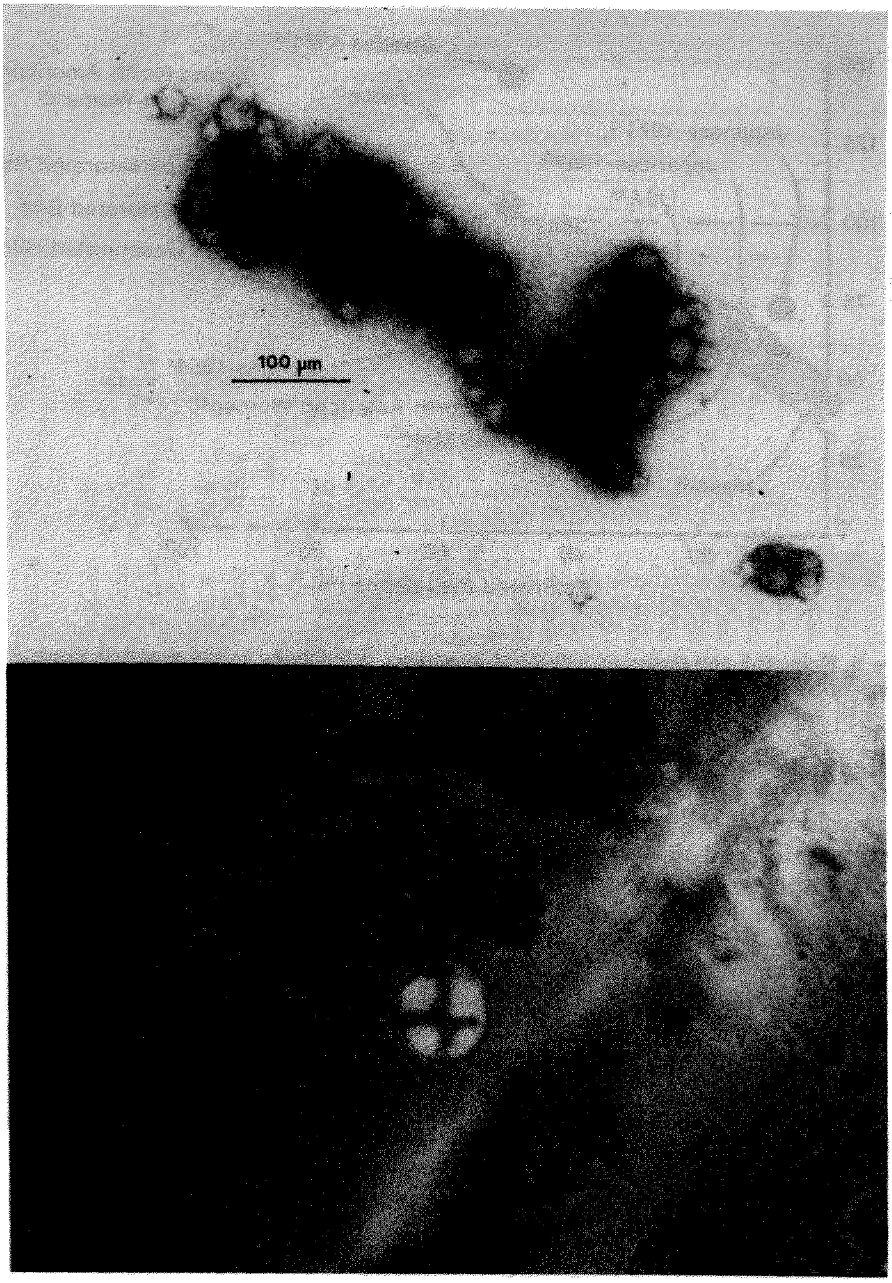

**Figure 4** (a) Aggregated unilamellar vesicles seen by electron microscopy. (b) Multilamellar vesicle measuring about 1000nm shown by polarizing light microscopy. The dark cross-like configuration on the vesicle is referred to as a Maltese cross and is characteristic.

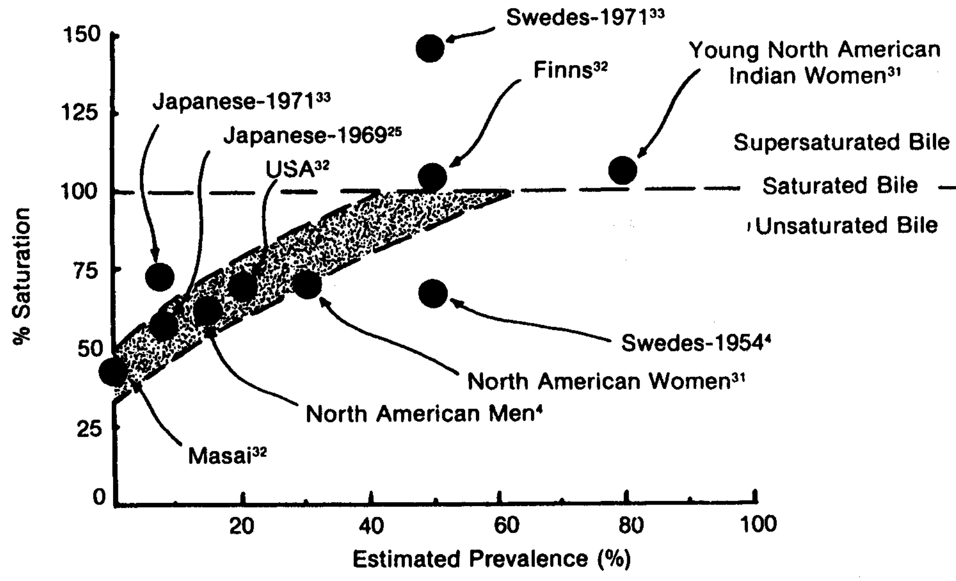

**Figure 5** Estimated prevalence of gallstones in various populations versus reported saturation of gallbladder bile from the same group. (From reference 40)

reduced bile salt pool in patients with stones[47]. Bile also tends to become supersaturated with age. This was first shown in the Chilean population[48] by Valdevesio *et al.* (Figure 6). Studies from Sweden have shown that the bile salt secretion rate, and the bile salt pool diminish with age, while the cholesterol secretion rate goes up [49]. These findings probably account for the increasing incidence of cholesterol gallstones with age. Estrogens promote cholesterol secretion into bile[50], and it seems likely that this is an important factor leading to supersaturation of bile with cholesterol in females and the preponderance of gallstones in females. Diet certainly affects bile composition[51–53]. Vegetarians rarely form cholesterol gallstones[54]. A variety of dietary causes have been proposed[51–53]. An infrequent cause of supersaturation is intestinal loss of the bile salt pool beyond the capacity of the hepatic synthesis to compensate as may be seen following ileal resection or diseases such as regional ileitis[55,56].

Bile does not remain supersaturated throughout the day. After meals when the bile salt pool is cycling, hepatic bile is much less saturated than during the interdigestive phase, especially the long interdigestive phase which occurs at night[57–60] (Figure 7). It is also during this period that the greatest degree of dehydration of bile in the gallbladder may occur.

### *Nucleation and Cholesterol Gallstone Formation*

While supersaturation is very common only a much smaller group of individuals develop crystals in their bile or go on to form stones. A key observation in this regard was made by Holan and Holzbach[42] who noted that gallbladder bile from

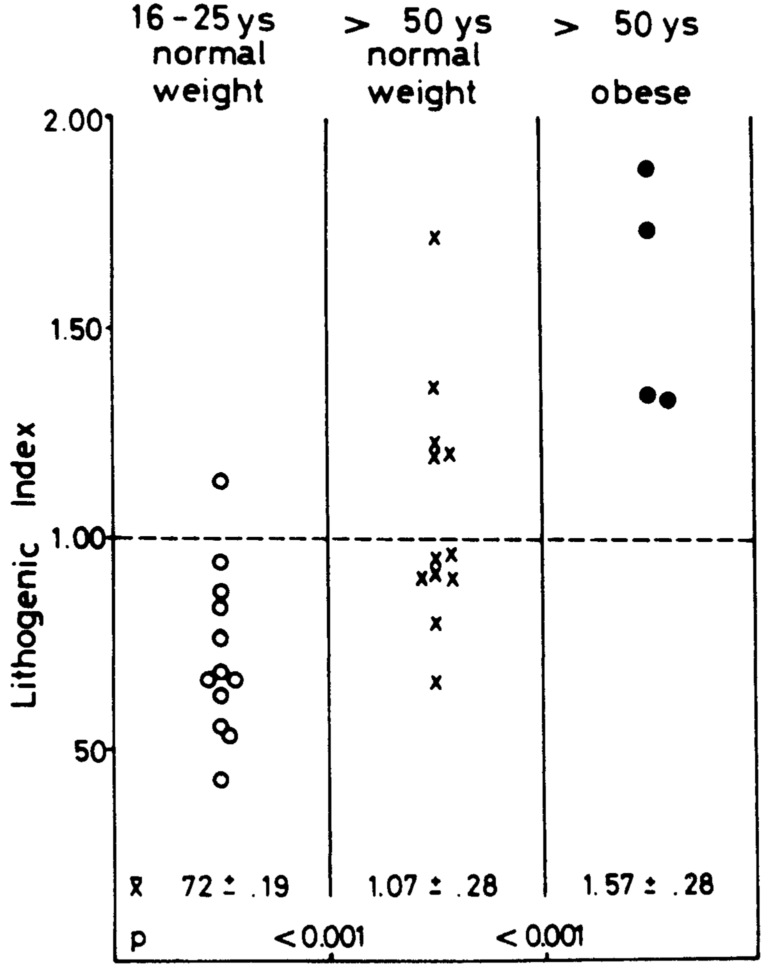

**Figure 6** Lithogenic index in patients above and below 50 years of age in Chile. The lithogenic index is similar to the cholesterol saturation index in biles with total lipid concentration greater than 2gm/dl as in this case. (From reference 48)

patients with cholesterol gallstones nucleated cholesterol crystals much more rapidly than equally supersaturated biles from patients without stones (Figure 8). They introduced a test called the "nucleation time test" which measured the time required for the appearance of cholesterol monohydrate crystals in a sample of bile which had been initially cleared of crystals by ultracentrifugation. This test remains very useful for examining the effects of proposed pronucleating and antinucleating factors in bile, but the term "nucleation time" is misleading since it implies detection of the unit crystal, which is much too small to see. The test detects crystals many orders of magnitude greater than the unit crystal; therefore it is measuring crystal growth as well as nucleation time, and a more appropriate term is "crystal observation time".

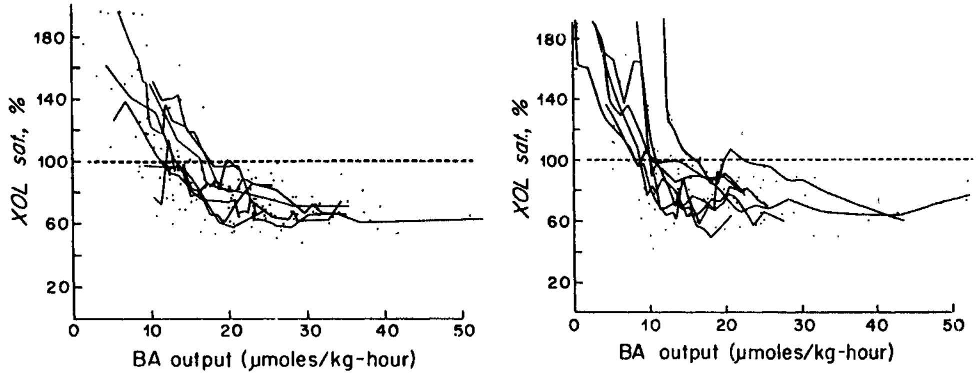

**Figure 7** Relationship between bile salt secretion and saturation of bile in patients with gallstones (left) and control patients (right). Low bile salt secretion rates occur at night and between meals when most of the bile salt pool resides in the gallbladder. (From reference 58)

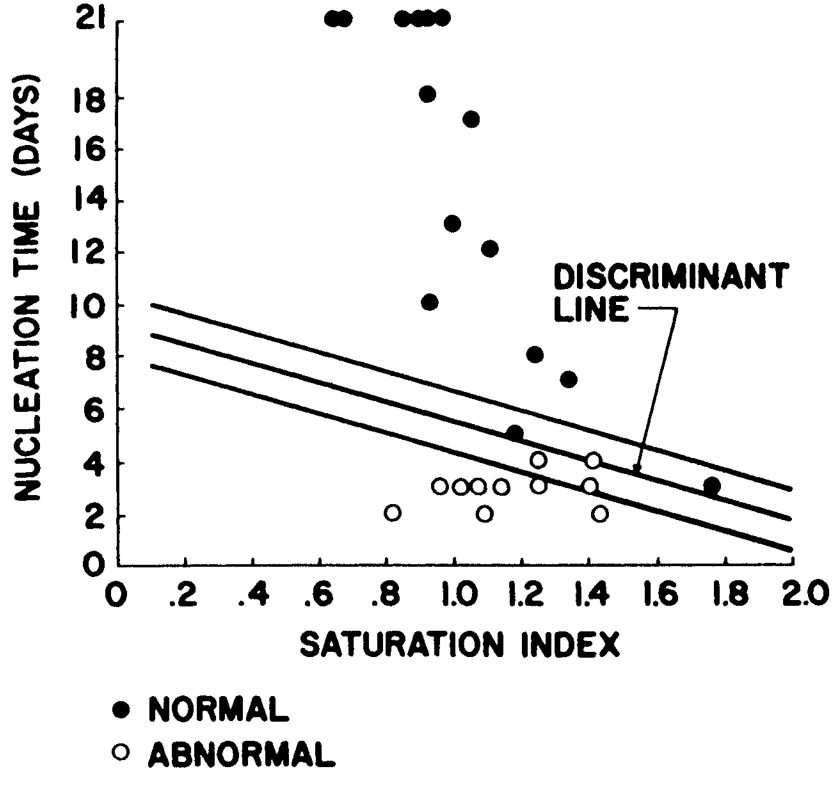

**Figure 8** Nucleation time versus cholesterol saturation index in gallbladder biles of patients with cholesterol gallstones (open circles) and in control patients (closed circles). Note that stone patients' biles nucleate faster despite having similar cholesterol saturation. (From reference 42)

Bile contains a variety of substances which influence precipitation of cholesterol from supersaturated solution, either promoting it (pronucleating factors) or retarding it (antinucleating factors). Several years ago we showed that bile from cholesterol gallstone patients contains a potent pronucleating factor[61] and much recent work has been done in this area in an attempt to identify the nature of that substance. Antinucleating factors have also been found in bile.

### *Pronucleating Substances*

### *Mucous glycoproteins*

Mucous glycoproteins, are a subgroup of glycoproteins with a high carbohydrate content (over 70% by weight) which contributes substantially to their high molecular weight (over $10^6$). Mucous glycoproteins are distinguished from serum glycoproteins by their low mannose content, and from proteoglycans by lack of uronic acid. Mucous glycoproteins were implicated in stone formation by the observation that stones contained a mucopolysaccharide matrix[62–64]. Subsequently, it was found that some lithogenic diets caused hypersecretion of mucous glycoprotein in the gallbladder prior to stone formation in animals[65–68]. Aspirin inhibited hypersecretion of mucous glycoprotein in the prairie dog model and prevented gallstone production without decreasing cholesterol saturation[69]. These studies suggested that mucous glycoprotein might be a pronucleator.

Lee *et al.*[68] directly examined the effect of mucous glycoprotein on nucleation. Human gallbladder mucin obtained from gallbladder mucosal scrapings accelerated the nucleation of cholesterol crystals when added to saturated prairie dog hepatic bile. Subsequently, it was shown that human gallbladder mucous glycoprotein accelerates the nucleation time of model bile systems[70] as well as control human gallbladder biles[71].

While there is no doubt that mucous glycoproteins are pronucleators, it is much less certain that either a quantitative or a qualitative abnormality of these compounds is the nucleation defect responsible for cholesterol gallstone formation. Lee *et al.*[72] found a significant increase in the gallbladder mucous glycoprotein concentration in patients with cholesterol gallstones compared to control patients, particularly if sludge was present. On the other hand we found no significant difference in the gallbladder mucous glycoprotein concentration between the two patient groups[73]. Furthermore, in qualitative studies, both the amino acid and carbohydrate content of mucous glycoproteins purified from patients with and without cholesterol gallstones were shown to be very similar[72,73]. The only reported difference in mucous glycoprotein between groups is in sulphate content[72,74]. Finally we showed that the mucous glycoprotein purified from normal control bile was just as effective in accelerating the nucleation time as mucous glycoprotein obtained from stone former bile[71].

### *Proteins other than mucous glycoproteins*

Early investigations in our laboratory showed that patients with cholesterol gallstones contained a potent nucleating factor that was not removed by steps which eliminated mucous glycoprotein[61]. Purified preparations of biliary proteins were prepared,removing only the mucous glycoproteins. These proteins were used to perform an experiment very similar to the one described above for mucous glycoproteins, i.e. gallbladder proteins obtained from patients with stones and controls without stones were added in identical amounts to control gallbladder bile[75]. These results are shown in Figure 9. We found that the proteins from patients with cholesterol gallstones were better pronucleators than the proteins of control patients. All protein samples from gallstone patients accelerated nucleation of control bile, but control patient proteins did so infrequently.

Further purification of the pronucleating protein has been achieved by using Concanavalin A affinity chromatography of hepatic bile samples[76]. Nucleation promoting activity was also reported to be present in patients without cholesterol gallstones but is less potent. This Con A binding glycoprotein has been shown to accelerate the nucleation time of model biles in a dose response manner (Figure 10)[77]. The number of crystals observed per day was also greater in models containing the higher concentration of Con A binding glycoprotein. Since mucous glycoprotein does not bind to Con A these results support the evidence that a non mucous glycoprotein may have an important role in the formation of cholesterol gallstones. The glycoprotein must be rich in mannose and/or glucose since it binds to Con A. Subsequently Groen *et al.* employing gel filtration chromatography with a Superose 12 column have found that nucleation promoting activity eluted in two peaks with molecular weights of 150 + /–30 kDa and < 5 kDa respectively. The < 5 kDa protein is believed to be a degraded product of the 150 kDa protein[78]. The identity of the 150 kDa glycoprotein remains to be determined. Our own most recent studies suggest that the pronucleating protein is of larger molecular weight and that it is probably an immunoglobulin[79].

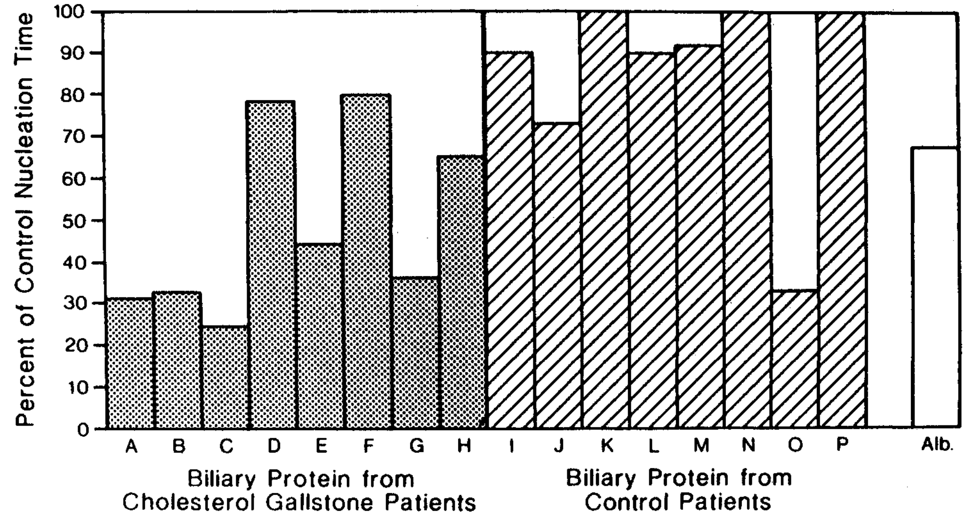

**Figure 9** Reduction in nucleation time of control slowly nucleating bile produced by the addition of purified gallbladder bile protein from stone patients or controls. The mass of protein added was the same in all studies. Note that the protein from the gallstone patients reduced the nucleation time. (From reference 75)

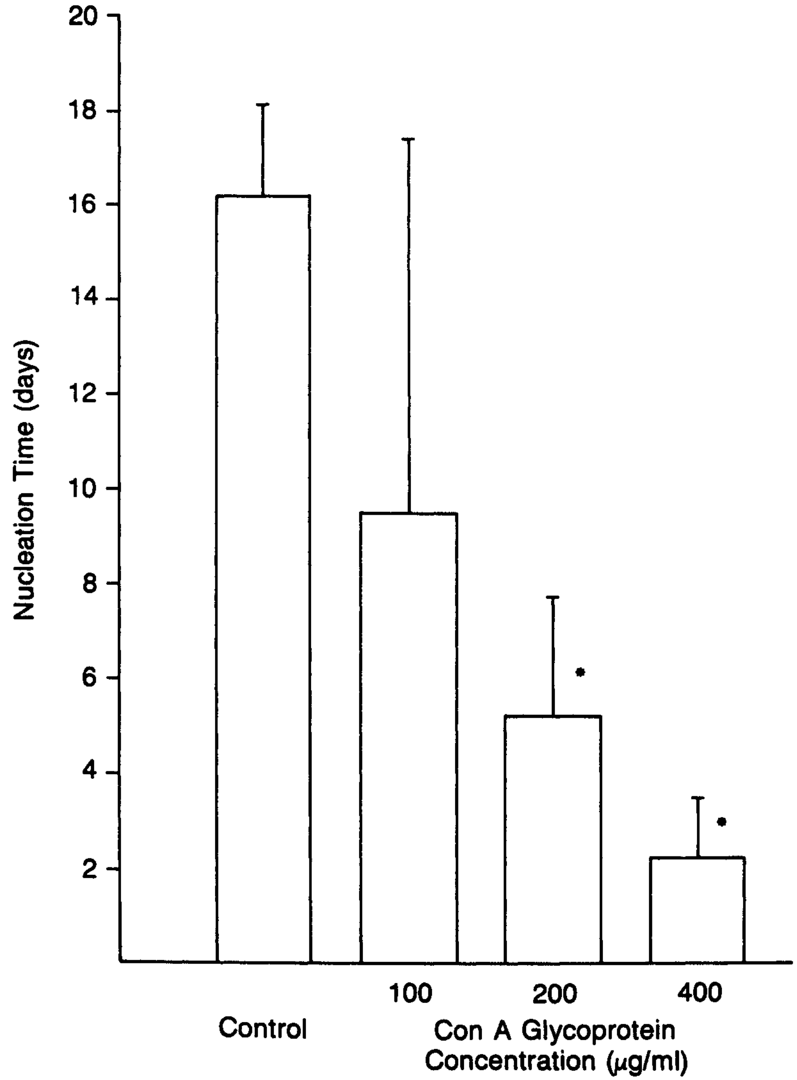

**Figure 10** Effect of Con A glycoprotein on nucleation time. (From reference 77)

*Other pronucleating substances*

Calcium and bilirubin have been proposed to be pronucleating substances. A common observation is that the centre of all cholesterol stones is pigmented and it has been shown that there is calcium as well as other inorganic substances in the centre of stones[80,81]. Calcium bilirubinate does not appear to accelerate nucleation[82], and the removal of calcium by chelation did not retard nucleation[82]. However, addition of calcium did appear to accelerate nucleation in a model bile system[35]. Recently it has been shown that the addition of calcium to human gallbladder bile does not accelerate the nucleation time but the number of crystals formed was increased[83]. Calcium does promote aggregation and fusion of certain types of phospholipid vesicles, although not phosphatidyl choline vesicles[84].

*Antinucleating substances*

*Apolipoproteins*

The nucleation time of model bile is more rapid than that of equally supersaturated bile obtained from normal persons[85]. This observation suggested that normal bile contained stabilizing antinucleating factors. Holzbach *et al.* determined that these nucleation inhibiting factors were bile proteins; detergent delipidated biliary proteins isolated from the bile of stone-free individuals prolonged the nucleation time when added to model bile[85]. Preincubation of these bile proteins with pronase destroyed the nucleation inhibiting effect. This was the first study implicating biliary proteins as cholesterol crystal nucleation inhibitors. A preliminary attempt to isolate and identify the anti-nucleating protein by gel filtration showed that the major antinucleating activity eluted in the included volume of the column. Whether proteins which co-eluted with the biliary lipids were associated with the lipids or eluted with lipids due to similar molecular size, was not determined. The most likely explanation however, as suggested by Holzbach *et al.* is that these proteins interact with bile lipids possibly forming complexes similar to the lipoproteins of serum[85].

Support for the role of apolipoproteins came from the work of Sewell *et al.*[86] and Ohisalo *et al.*[87] who demonstrated their presence in human gallbladder bile. Subsequently, purified serum apolipoproteins, namely apoprotein A–1, A–2, and C–3, were added to model biles to study their effect on nucleation time[88]. Apoprotein A–1 and A–2 inhibited the rate of formation of cholesterol crystals in supersaturated model bile systems. Apoprotein C–3 had no effect. Furthermore, Apoprotein A–1 and A–2 were identified in the included gel filtration fraction shown previously to contain the cholesterol nucleation inhibiting factor[88]. These results strongly suggest that Apoproteins A–1 and A–2 are proteins that inhibit cholesterol crystal nucleation in human supersaturated gallbladder bile. One might expect then that the concentration of these proteins would be higher in patients without gallstones. However, this was not found to be so. The concentrations of Apoprotein A–1 and A–2 is similar in patients with and without gallstones[86].

*Other possible nucleation inhibiting proteins*

Groen *et al.* recently reported nucleation inhibiting activity in human bile in the unbound protein fraction from a Concanavalin A Sepharose lectin column[76]. The nucleation inhibiting fraction was unstable; inhibiting activity was lost after 24 hr at 37°C. This nucleating inhibiting protein may be Apolipoprotein A–1, which does not contain carbohydrates[89], and would not bind to Con A. The activity of this factor is transient at 37°C likely because it is denatured by endogenous proteolytic enzymes in gallbladder bile[76].

Holzbach's group has used several lectins in order to isolate nucleating and anti-nucleating proteins from normal gallbladder bile[90]. The protein fraction isolated by Helix pomatia, a lectin specific for N-acetyl-D-galactosamine[91] inhibited both nucleation and crystal growth. This protein fraction was reported to have greater potency than Apolipoprotein A–1. The identity of this factor remains to be determined.

*Mode of action of pronucleating and antinucleating substances*

There are at least four mechanisms for altering the rate of appearance of crystals. First the rate of maturation of biliary vesicles could be altered, accelerating or delaying the production of vesicles with a high cholesterol:phospholipid ratio. There is some evidence that the Con A pronucleating protein acts in this way[92]. Nucleation rates from such mature vesicles could also be altered by substances promoting aggregation or fusion of vesicles. Certain proteins as well as other substances have been implicated as aggregating agents[93–96] or fusogens[97–101], and the biliary proteins which are being described as pronucleating or antinucleating could act in this way. Finally the agents could act by accelerating or slowing crystal growth.

### *From Crystal to Stone — Stasis and Glue*

Crystalbilia does not necessarily lead to stones any more than supersaturation leads to crystalbilia. Although there are many aspects of supersaturation and crystal formation yet to be understood, nowhere is knowledge more lacking than in the processes which lead from crystals to stones. There are two guiding facts. Stones are complex structures consisting of a matrix and inorganic salts as well as cholesterol and these non-cholesterol materials may have the role of binding the stone together. Secondly, crystals are excreted from the gallbladder, so for stones to form there must be a period of crystal retention, i.e. stasis.

There are multiple lines of evidence that abnormalities in motility are important in stone formation. There is an increased incidence of stones in situations where stasis occurs, such as pregnancy[102], treatment with female sex hormones[102,103], during fasting[104,105], and in patients on total parenteral nutrition (TPN)[104,106]. Stasis may also contribute to stone formation after vagotomy[107,108].

TPN associated stone formation may be due to stasis secondary to decreased gallbladder stimulation. Patients receiving some oral supplementation have a much lower incidence of stone formation than those who receive no oral intake[104,109]. Although such stones may be cholesterol in type, they are usually pigment stones[104].

The increased risk of gallstones in multiparous females[110] may be due in part to gallbladder stasis. Gallbladder emptying is impaired in the third trimester of pregnancy when progesterone levels are highest[102,110–112]. It has been found that pregnant women and women taking contraceptive steroids have increased gallbladder volumes[111]. In female guinea pigs, gallbladder contractile response to CCK is related to the number of progesterone receptors in the gallbladder wall[113], with maximum contractility occurring after oophorectomy when progesterone levels are lowest. Conversely, progesterone pretreatment decreases the response to CCK or acetylcholine[114,115]. In pregnant guinea pigs the response to CCK is reduced whereas the response to extracellular potassium chloride is unaffected[116], this suggests that the effect of sex hormones is at the receptor level and that the gallbladder muscle is itself unimpaired.

There is now strong evidence that the composition of the bile may affect gallbladder motility, and particularly that high cholesterol concentrations reduce motility by a direct non-receptor mediated effect on the muscle of the gallbladder.

Behar has recently shown that human gallbladders exposed to a high cholesterol environment have decreased contractility when tested *in vitro*[117], and it has been known for some time that impaired contractility precedes the development of stones in animals fed a high cholesterol diet[116]. This impairment is present even when agents which stimulate muscle are used, indicating that the problem is at the level of the muscle cell. The means by which contractility is impaired by high levels of cholesterol is unknown. Cholesterol is incorporated into cell membranes, and can affect membrane fluidity[118,119] and perhaps the operation of ion channels, but whether this is the mechanism whereby contraction is impaired is presently unknown.

Prostaglandins have been implicated as the mediators of dysmotility in some studies[120]. Prostaglandins are stimulators of biliary smooth muscle[120–123], and the early increase in cystic duct resistance induced by a high cholesterol lithogenic diet correlated with PGE concentration and PGFalpha synthesis in the gallbladder[124]. Some authors have shown a biphasic response to prostaglandins with initial increased motility followed by a decrease in motility[121,125,126], and it has been suggested that impairment of emptying may result from superimposed changes in both gallbladder contractility and cystic duct resistance.

Direct measurements of gallbladder emptying in humans with gallstones have produced somewhat conflicting results. Shaffer reported a subgroup of patients with decreased motility[127], but this study also implies that many patients develop stones without an impairment in motility. Such studies are difficult to evaluate because the stimulus to contraction is exogenous. The effect of stasis is to give time for such kinetic events as maturation, nucleation and crystal growth to occur, as well as to allow for retention of crystals in the gallbladder. Stasis also gives extra time for concentration of bile to occur. As noted above, we have found crystal formation to be associated with high total lipid and protein concentrations in the gallbladder[36], and this could be brought about by stasis. Stasis appears to result in the formation of sludge. Sludge is a complex precipitate consisting mainly of calcium bilirubinate and mucous glycoprotein. However, sludge may also contain cholesterol crystals[128], and some patients who developed sludge have gone on to form cholesterol gallstones[128]. Sludge is an ultrasonic finding and may not always be present when crystals are in bile. We have found that persons without ultrasonic evidence of sludge may have many cholesterol crystals in bile[36]; such bile is frequently very high in total lipid concentration suggesting prolonged residence in gallbladder, i.e. stasis[36]. It should also be remembered that there are zones or layers in the gallbladder containing bile of different concentration, i.e. bile can separate into elements of different density in the gallbladder[129]. Certain of these zones may not exchange in the same way as others, forming areas of stasis in a gallbladder that is otherwise functioning normally in terms of contraction.

Cholesterol crystals left in a test tube will not form a stone. At most, loose aggregates of several hundred crystals form. At present, little can be said about how the disparate elements of a stone come together or why some persons form a single stone and others form multiple stones. The scaffolding matrix of mucous glycoprotein would appear to be essential. Mucous glycoprotein is in solution in the bulk phase of bile, i.e. in the centre of the gallbladder, but on the wall it is in gel form. It is possible that the earliest assembly of a stone takes place in this area. As noted above, the centre of most cholesterol gallstones contains calcium bilirubinate, and it is possible that this substance acts as an agglomerating agent for cholesterol crystals at an early stage of stone formation. Many more studies are required to understand these early events in stone formation.

### *References*

1. Barbara, L. (1984) Epidemiology of gallstones disease: "Sirmione Study". In Epidemiology and prevention of gallstone disease. L. Capocaccia, G. Ricci, F. Angelico, M. Angelico, and A.F. Attili. Editors. MTP Press pp. 23–25
2. Covarrubias, C., Valdivieso, V., and Nervi, F. (1984) Epidemiology of gallstone disease in Chile. In Epidemiology and prevention of gallstone disease. L. Capocaccia, G. Ricci, F. Angelico, M. Angelico, and A.F. Attili. Editors. MTP Press pp. 26–30
3. Chijiiwa, K. and Nagai, M. (1989) Bile salt micelle can sustain more cholesterol in the intermicellar aqueous phase than the maximal aqueous solubility. *Archives of Biochem and Biophys.* **270**, 472–477
4. Miettinen, T., Kesaniemi, Y.A., Jarvinen, H. *et al.* (1986) Cholesterol precursor sterols, and cholestanol in human bile and gallstones. *Gastroenterology* **90**, 858–864
5. Angelico, M., Alvaro, D., Attili, A.F. (1985) Mechanisms of secretion of biliary phosphatidyl cholines: the role of bile acids. *Ital. J. Gastroent.* **17**, 278–281
6. Cantafora, A., Di Biase, A., Alvaro, D., Angelico, M., Marin, M., and Attili, A.F. (1983) High performance liquid chromatographic analysis of molecular species of phosphatidylcholine — development of quantitative assay and its application to human bile. *Clin. Chem. Acta* **134**, 281–295
7. Ahlberg, J., Curstedt, T., Einarsson, K., Sjovall, J. (1981) Molecular species of biliary phosphatidylcholines in gallstone patients: the influence of treatment with cholic acid and chenodeoxycholic acid. *J. Lipid. Res.* **22**, 404–409
8. Mazer, N.A., Benedek, G.B. and Carey, M.C. (1980) Quasielastic light-scattering studies of aqueous biliary lipid systems. Mixed micelle formation in bile salt-lecithin solutions. *Biochemistry* **19**, 601–615
9. Donovan, J.M. and Carey, M.C. (1990) Cholesterol "carriers" in bile. Hepatology in press
10. Entenman, C., Holloway, R.J., Albright, M.C., Leong, G. (1968) Bile acids and lipid metabolism. I. Stimulation of bile lipid excretion by various bile acids. *Proc. Soc. Exp. Med.* **127**, 1003–1006
11. Nillson, S., Schersten, T. (1969) Importance of bile acids for phospholipid secretion into human hepatic bile. *Gastroenterology* **57**: 525–532
12. Wagner, C.I., Trotman, B.W, Soloway, R. (1976) Kinetic analysis of biliary lipid excretion in man and dog. *J. Clin. Invest.* **57**, 473–477
13. Hardison, W.G.M., Apter, J.T. (1972) Micellar theory of biliary cholesterol excretion. *Am. J. Physiol.* **22**, 61–67
14. Evans, W.H., Kremmer, T., Culvenor, J.G. (1976) Role of membranes in bile formation. Comparison of the composition of bile and a liver bile-canalicular plasma membrane subfraction. *Biochem. J.* **154**, 589–595
15. Mazer, N.A., Schurtenberger, P., Carey, M.C., Preisig, R., Weigand, K., Kanzig, W. (1984) Quasi-elastic light scattering of native hepatic bile from the dog: comparison with aggregative behavior of model biliary lipid systems. *Biochemistry* **23**, 1994–2005
16. Somjen, G.J., Gilat, T. (1983) A non-micellar mode of cholesterol transport in human bile. *FEBS* **156**, 265–268
17. Somjen, G.J., Gilat, T. (1985) Contribution of vesicular and micellar carriers to cholesterol transport in human bile. *J. Lipid. Res.* **26**, 699–704
18. Ulloa, N., Garrido, J., Nervi, F. (1987) Ultracentrifugal isolation of vesicular carriers of biliary cholesterol in native human and rat bile. *Hepatology* **7**: 235–244
19. Cohen, D.E., Angelico, M. and Carey, M.C. (1989) Quasielastic light scattering evidence for vesicular secretion of biliary lipids. *Am. J. Physiol.* **257**, G1–G8
20. Balint, J.A., Beeler, D.A., Kryriakides, E.C., Treble, D.H. (1971) The effect of bile salts upon lecithin synthesis. *J. Lab. Clin. Med.* **77**, 122–131
21. Turley, S.D., Dietschy, J.M. (1988) The metabolism and excretion of cholesterol by the liver. In Arias, I.M., Jakoby, W.B., Popper, H. *et al.* (1988) Eds. The Liver: Biology and Pathobiology, pp. 617–641.New York: Raven Press Ltd
22. Coleman, R. (1987) Biochemistry of bile secretion. *Biochem. J.* **244**: 249–261

23. Barnwell, S.G., Lowe, P.J., Coleman, R. (1984) The effects of colchicine on secretion into bile of bile salts, phospholipids, cholesterol and plasma membrane enzymes: bile salts are secreted unaccompanied by phospholipids and cholesterol. *Biochem. J.* **220**, 723–731
24. Simion, F.A., Fleisher, B.F., Fleisher, S. (1984) Subcellular distribution of bile acids, bile salts and taurocholate binding sites in rat liver. *Biochemistry* **22**, 6459–6466
25. Lowe, P.J., Barnwell, S.G., Coleman, R. (1984) Rapid kinetic analysis of the bile salt dependent secretion of phospholipid cholesterol and a plasma membrane enzyme into bile. *Biochem. J.* **222**, 631–637
26. Dumont, M., Erlinger, S. (1977) Diminution de la secretion du cholesterol et des phospholipids dans la bile sous l'influence de colorants cholephiles chez le hamster. *Gastroenterol. Clin. Biol.* **1**, 891–896
27. Kuipers, F., Verkade, H.J., Wolbers, M.J., Havinga, R., Vonk, R.J. (1989) The uncoupling of biliary lipid from bile acid secretion by organic anions in the rat. Presented at International Lugano Symposium on Biliary Physiology and Diseases: Strategies for the treatment of hepatobiliary diseases. Falk Symposium No. 53
28. Nervi, F., Marinovic, I., Rigotti, A., Ulloa, N. (1988) Regulation of biliary cholesterol secretion: Functional relationship between the canalicular and sinusoidal cholesterol secretory pathways in the rat. *J. Clin. Invest.* **82**, 1818–1825
29. Ruetz, S., Fricker, G., Hugentobler, G., Winterhalter, K., Kurz, G., Meir, P.J. (1987) Isolation and characterization of the putative canalicular bile salt transport system of rat liver. *J. Biol. Chem.* **262**, 11324–11330
30. Collins, J.J., Phillips, M.C., (1982) The stability and structure of cholesterol rich codispersions of cholesterol and phosphatidylcholine. *J. Lipid. Res.* **23**, 291–298
31. Harvey, P.R.C., Somjen, G., Lichtenberg, M.S., Petrunka, C., Gilat, T., Strasberg, S. (1987) Nucleation of cholesterol from vesicles isolated from bile of patients with and without cholesterol gallstones. *Biochim. Biophys. Acta.* **921**, 198–204
32. McLean, L.R. and Phillips, M.C. (1982) Cholesterol desorption from clusters of phosphatidylcholine and cholesterol in unilamellar vesicle bilayers during lipid transfer or exchange. *Biochemistry* **21**, 4053–4059
33. Nichols, J.W. (1988) Phospholipid transfer between phosphatidylcholine-taurocholate mixed micelles. *Biochemistry* **27** 3925–3931
34. Harvey, P.R.C., Somjen, G., Gilat, T., Gallinger, S., Strasberg, S. (1988) Vesicular cholesterol in bile. Relationship to protein concentration and nucleation time. *Biochim. Biophys. Acta.* **958**, 10–18
35. Kibe, A., Dudley, M.A., Halpern, Z., Lynn, M.P., Breuer, A.C., Holzbach, R.T. (1985) Factors affecting cholesterol monohydrate crystal nucleation time on model of systems of supersaturated bile. *J. Lipid. Res.* **26**, 1102–1111
36. Strasberg, S.M., Toth, J.L., Gallinger, S. and Harvey, P.R.C. (1990) High protein and total lipid concentration are associated with reduced metastability in an early stage of cholesterol gallstone formation. *Gastroenterology* **98**, 739–746
37. Halpern, Z., Dudley, M.A., Lynn, M.C., Nader, J.M., Breuer, A.C., Holzbach, R.T. (1986) Vesicle aggregation in model systems of supersaturated bile: relation to crystal nucleation and lipid composition of the vesicular phase. *J. Lipid. Res.* **27**, 295–306
38. Halpern, Z., Dudley, M.A., Kibe, A., Lynn, M.P., Breuer, A.C., Holzbach, R.T. (1986) Rapid vesicle formation and aggregation in abnormal human bile. *Gastroenterology* **90**, 875–885
39. Hui, S.W. (1988) The spatial distribution of cholesterol in membranes. In Biology of cholesterol. PL Yeagle ed. pp. 213–231.CRC Press, Boca Raton, Florida
40. Redinger, R.N. and Small, D.M. (1972) Bile composition, bile salt metabolism and gallstones. *Arch. Internal Med.* **130**, 618–630
41. Holzbach, R.T., Marsh, M., Olszewski, M.F. and Holan, K. (1973) Cholesterol solubility in bile: evidence that supersaturated bile is frequent in healthy man. *J. Clin. Invest.* **52**, 1467–1479
42. Holan, K.R., Holzbach, R.T., Hermann, R.E., Cooperman, A.M., Claffey, W.J. (1979) Nucleation time: a key factor in the pathogenesis of cholesterol gallstone disease. *Gastroenterology* **77**, 611–617
43. Gollish, S.H., Burnstein, M.J., Ilson, R.G., Petrunka, C.N. and Strasberg, S.M. (1983) Nucleation of cholesterol monohydrate crystals from hepatic and gallbladder bile of patients with cholesterol gallstones. *Gut* **24**, 836–844
44. Shaffer, E.A. and Small, D.M. (1977) Biliary lipid secretion in cholesterol gallstone disease: the effect of cholecystectomy and obesity. *J. Clin. Invest.* **59**, 828–840
45. Grundy, S.M., Duane, W.C., Adler, R.D., Aron, J.M., Metzger, A.L. (1974) Biliary lipid outputs in young women with cholesterol gallstones. *Metabolism* **23**, 67–73

46. Bennion, L.J. and Grundy, S.M. (1975) Effects of obesity and caloric intake on biliary lipid metabolism in man. *J. Clin. Invest.* **56**, 996–1011
47. Vlahcevic, Z.R., Bell, C.C.J., Buhac, I., Farrar, J.T. and Swell, L. (1970) Diminished bile acid pool in patients with gallstones. *Gastroenterology* **59**, 165–173
48. Valdivieso, V., Palma, R., Wunkhaus, R., Antezana, C., Severin, C., and Contreras, A. (1978) Effect of aging on biliary lipid composition and bile acid metabolism in normal Chilean women. *Gastroenterology* **74**, 871–874
49. Einarsson, K., Nilsell, K., Leijd, B., and Angelin, B. (1985) Influence of age on secretion of cholesterol and synthesis of bile acids by the liver. *N. Engl. J. Med.* **313**, 277–282
50. Bennion, L.J., Mott, D.M., and Howard, B.V. (1980) Oral contraceptives raise the cholesterol saturation of bile by increasing biliary cholesterol secretion. *Metabolism* **29**, 18–22
51. Pomare, E.W., Heaton, K.W., Low-Beer, T.S. and Espiner, H.J. (1976) The effect of wheat bran upon bile salt metabolism and upon the lipid composition of bile in gallstone patients. *Dig. Dis.* **21**, 521–526
52. Nervi, F., Covarrubias, C., Bravo, P., Velasco, N., Ulloa, N., Cruz, F., Fava, M., Severin, C., Del Pozo, R., Antezana, C., Valdivieso, V. and Arteaga, A. (1989) Influence of legume intake on biliary lipids and cholesterol saturation in young Chilean men. *Gastroenterology* **96**, 825–830
53. Low-Beer, T.S. (1985) Nutrition and cholesterol gallstones. *Proceedings Nutrition Soc.* **44**, 127–134
54. Pixley, F., Wilson, D., McPherson, K. and Mann, J. (1985) Effect of vegetarianism on development of gallstones in women. *Br. Med. J.* **291**, 11–12
55. Heaton, K.W., Austad, W.I., Lack, I., Tyor, M.P., (1968) Enterohepatic circulation of $^{14}$C-labelled bile salts in disorders of the distal small bowel. *Gastroenterology* **55**, 5–16
56. Dowling, R.H., Bell, G.D., White, J. (1972) Lithogenic bile in patients with ileal dysfunction. *Gut* **13**, 415–420
57. Northfield, T.C. and Hoffman, A.F. (1973) Biliary lipid secretion in gallstone patients. *Lancet* **1**, 747–748
58. Northfield, T.C. and Hoffman, A.F. (1975) Biliary lipid output during three meals and an overnight fast. *Gut* **16**, 1–17
59. Kupfer, R.M. and Northfield, T.C. (1983) Diurnal variation in cholesterol saturation of gallbladder bile. *Gut* **24**, 950–953
60. Metzger, A.L., Adler, R., Heymsfield, S., and Grundy, S.M. (1973) Diurnal variation in biliary lipid composition. Possible role in cholesterol gallstone formation. *N. Engl. J. Med.* **288**, 333–336
61. Burnstein, M.J., Ilson, R.G., Petrunka, C.N., Taylor, R.D. and Strasberg, S.M. (1983) Evidence for a potent nucleating factor in the gallbladder bile of patients with cholesterol gallstones. *Gastroenterology* **85**, 801–807
62. Womack, N.A., Zeppa, R., Irvin, G.L. (1963) The anatomy of gallstones. *Ann. Surg.* **157**, 670–686
63. Hulten, O. (1970) Contractions of the gallbladder and the formation of gallstones. *Acta. Chir. Scand.* **136**, 53–56
64. Maki, T., Matsushiro, T., Suzuki, N., Nakamura, N. (1971) Role of sulfated glycoprotein in gallstone formation. *Surg. Gynecol. Obst.* **132**, 846–854
65. Freston, J.W., Bouchier, I.A.D., Newman, J. (1969) Biliary mucous substances in dihydrocholesterol-induced cholelithiasis. *Gastroenterology* **57**, 670–678
66. Womack, N.A. (1971— The development of gallstones. *Surg. Gynecol. Obst.* **133**, 937–945
67. Lee, S.P. (1981) Hypersecretion of mucus glycoprotein by the gallbladder epithelium in experimental cholelithiasis. *J. Pathology* **134**, 199–207
68. Lee, S.P., Lamont, J.T., Carey, M.C. (1981) Role of gallbladder mucus hypersecretion in the evolution of cholesterol gallstones. *J. Clin. Invest,* **67**, 1712–1723
69. Lee, S.P., Carey, M.C., LaMont, J.T. (1981) Aspirin prevention of cholesterol gallstone formation in prairie dogs. *Science* **211**, 1429–1431
70. Levy, P.F., Smith, B.F., LaMont, J.T. (1984) Human gallbladder mucin accelerates nucleation of cholesterol in artificial bile. *Gastroenterology* **87**, 270–275
71. Gallinger, S., Taylor, R.D., Harvey, P.R.C., Petrunka, C.N., Strasberg, S.M. (1985) Effect of mucous glycoprotein on nucleation time of human bile. *Gastroenterology* **89**, 648–658
72. Lee, S.P., Nicholls, J.F., (1986) Nature and composition of biliary sludge. *Gastroenterology* **90**, 677–686
73. Harvey, P.R.C., Rupar, C.A. Gallinger, S., Petrunka, C.N., Strasberg, S.M. (1986) Quantitative and qualitative comparison of gallbladder mucous glycoprotein from patients with and without gallstones. *Gut* **27**, 374–381

74. Maki, T., Matsushiro, T., Suzuki, N., Nakamura, N. (1971) Role of sulfated glycoproteins in gallstone formation. *Surg. Gynecol. Obst.* **132**, 846–854
75. Gallinger, S., Harvey, P.R.C., Petrunka, C.N. Ilson, R.G., Strasberg, S.M. (1987) Biliary proteins and the nucleation defect in cholesterol cholelithiasis. *Gastroenterology* **92**, 867–875
76. Groen, A.K., Stout, J.P.J., Drapers, J.A.G., Hoek, J.F., Grijm, R., Tytgat, G.N.J. (1988) Cholesterol influencing activity in T-tube bile. *Hepatology* **8**, 347–352
77. Harvey, P.R.C., Upadhya, A, Toth, J.L. and Strasberg, S.M. (1989) Lectin binding characteristics of a cholesterol nucleation promoting protein. *Clin. Chim. Acta.* **185**, 185–190
78. Groen, A.K., Stout, J.P.J., Noordam, C., Korsen, A., Hoek, F.J., Jansen, P.L.M., Tytgat, G.N.J. (1987) Isolation and partial characterization of a cholesterol nucleation promoting factor from human bile. *Hepatology* **7**, 1023 (Abstr).
79. Harvey, P.R.C., Upadhya, G.A., and Strasberg, S.M. (1989) Immunoglobulins as nucleating agents in gallbladder bile. *Hepatology* **10**, 601 (Abstr).
80. Been, J.M., Bills, P.M., and Lewis, D. (1979) Microstructure of gallstones. *Gastroenterology* **76**, 548–555
81. Pitchumoni, C.S., Viswanathan, K.V., and Moore, E.W. (1987) Analysis and localization of elements in human cholesterol gallstones: Calcium and other elements are present in the central (nidus) region. *Gastroenterology* **92**, 1764 (abstract).
82. Gallinger, S., Harvey, P.R.C., Petrunka, C.N., and Strasberg, S.M. (1986) Effect of ionised calcium on the *in vitro* nucleation of cholesterol and calcium bilirubinate in human gallbladder bile. *Gut* **27**, 1382–1386
83. Neithercut, W.D. (1989) Effect of calcium, magnesium and sodium ions on *in vitro* nucleation of human gall bladder bile. *Gut* **30**, 665–670
84. Papahadjopoulos, D., Vail, W.J., Pangborn, W.A., Poste, G. (1976) Studies on membrane fusion II. Induction of fusion in pure phospholipid membranes by calcium ions and other divalent metals. *Biochim. Biophys. Acta.* **448**, 265–283
85. Holzbach, T.R., Kibe, A., Thiel, E., Howell, J.H., Marsh, M., Hermann, R.E. (1984) Biliary proteins: Unique inhibitors of cholesterol crystal nucleation in human gallbladder bile. *J. Clin. Invest.* **73**, 35–45
86. Sewell, R.B., Mao, S.J.T. Kawamoto,T., LaRusso, N.F. (1983) Apolipoproteins of high, low, and very low density lipoproteins in human bile. *J. Lipid Res.* **24**, 391–401
87. Ohisalo, J.J., Keso, L., Ehnholm, C. (1983) Apolipoproteins AI and AII in human bile. *Acta. Physiol. Scand.* **119**, 303–304
88. Kibe, A., Holzbach, R.T., LaRusso, N.F., Mao, S.J.T. (1984) Inhibition of cholesterol crystal formation by apolipoproteins in supersaturated model bile. *Science* **25**, 514–516
89. Mahley, R.W., Innerarity, T.L., Rall, S.C., Weisgraber, K.H. (1984) Plasma lipoproteins: apolipoprotein structure and function. *J. Lipid Res.* **25**, 1277–1294
90. Busch, N., Matiuck, N.V., Cottle, A., Mancuso, D.J., Tokumo, H., Holzbach, R.T. (1988) Inhibiting and promoting effects on cholesterol crystal nucleation and growth rate are found in protein fractions from normal human gallbladder bile. *Hepatology* **8**, 1224 (Abstr)
91. Goldstein, I.J., Hayes, C.E. (1978) The lectins: Carbohydrate-binding proteins of plants and animals. *Adv. Carbohydr. Chem. Biochem.* **35**, 127–340
92. Groen, A.K., Ottenhoff, R., Jansen, P.L.M., van Marle, J., Tytgat, G.N.J. (1989) Effect of cholesterol nucleation-promoting activity on cholesterol solubilization in model bile. *J. Lipid. Res.* **30**, 51–58
93. Gad, A.E., Eytan, G.D. (1983) Chlorophylls as probes for membrane fusion. Polymyxin B induced fusion of liposomes. *Biochim. Biophys. Acta.* **727**, 170–176
94. Morgan, C.G., Williamson, H., Fuller, S., Hudson, B. (1983) Melittin induces fusion of unilamellar phospholipid vesicles. *Biochim. Biophys. Acta.* **732**, 668–674
95. Gad, A.E., Silver, B.L., Eytan, G.D (1982) Polycation-induced fusion of negatively-charged vesicles. *Biochim. Biophys. Acta.* **690**, 124–132
96. Lampe, P.D., Wei, G.J., Nelsestuen, G.L. (1983) Stopped-flow studies of myelin basic protein association with phospholipid vesicles and subsequent vesicle aggregation. *Biochemistry* **22**, 1594–1599
97. White, J., Helenius, A., Gething, M.J. (1982) Haemagglutinin of influenza virus expressed from a cloned gene promotes membrane fusion. *Nature* **300**, 658–659
98. Lopez Vinals, A.E., Farias, R.N., Morero, R.D. (1987) Characterization of the fusogenic properties of glyceraldehyde-3-phosphate dehydrogenase: fusion of phospholipid vesicles. *Biochem. Biophys. Res. Comm.* **143**, 403–409

99. Schenkman, S., Soares de Araujo, P., Dijkman, R., Quina, F.H., Chaimovich, H. (1981) Effects of temperature and lipid composition on the serum albumin-induced aggregation and fusion of small unilamellar vesicles. *Biochim. Biophys. Acta.* **649**, 633-641
100. Farias, R.N., Vinals, A.L., Morero, R.D. (1985) Insulin-mediated fusion of negatively charged phospholipid vesicles at low pH. *Biochem. Biophys. Res. Comm.* **128**, 68–74
101. Cabiaux, V., Vandenbranden, M., Falmagne, P., Ruysschaert, J.M. (1984) Diphtheria toxin induces fusion of small unilamellar vesicles at low pH. *Biochim. Biophys. Acta.* **775**, 31–36
102. Braverman, D.Z., Johnson, M.L. Kern, F. (1980) Effects of pregnancy and contraceptive steroids on gallbladder function. *N. Engl. J. Med.* **302**, 362–364
103. Boston Collaborative Drug Surveillance Programme: (1974) Surgically confirmed gallbladder disease, venous thromboembolism and breast tumors in relation to postmenopausal estrogen therapy. *N. Engl. J. Med.* **290**, 15–18
104. Roslyn, J.J., Pitt, H.A., Mann, L.L., Ament, M.E., Den Besten, L. (1983) Gallbladder disease in patients on long-term parenteral. *Gastroenterology* **84**, 148–54
105. Maugdal, D.P., Kupfer, R.M., Zentler-Munro, P.L., Northfield, T.C. (1980) Postprandial gallbladder emptying in patient with gallstones. *Br. Med. J.* **1**, 141–2
106. Messing, B., Bories, C., Kunstlinger, F., Bernier, J.J. (1983) Does total parenteral nutrition induce gallbladder sludge formation and lithiasis? *Gastroenterology* **84**, 1012–19
107. Clave, R.A., Gaspar, M.R. (1969) Incidence of gallbladder disease after vagotomy. *Am. J. Surg.* **118**, 169–76
108. Sapala, M.A., Sapala, J.A., Resto Salto, A.D., Bouwman, D.L. (1979) Cholelithiasis following subtotal gastrectomy and vagotomy. *Surg. Gynecol. Obstet.* **148**, 36–8
109. Holzbach, R.T. (1983) Gallbladder stasis: consequence of long-term parenteral hyperalimentation and risk factor of cholelithiasis. *Gastroenterology* **84**, 1055–8
110. Friedman, G.D., Kannel, W.B., Dawber, T.R. (1966) The epidemiology of gallbladder disease: observation in the Framingham study. *J. Chronic. Dis.* **19**, 273–6
111. Everson, G.T., McKinley, C., Lawson, C., Johnson, M., Kern, F. Jr. (1982) Gallbladder function in the human female: effect of the ovulatory cycle, pregnancy and contraceptive steroids. *Gastroenterology* **82**, 711–15
112. Daignault, P.G., Fazekas, A.G., Mersereau, W.A., Fried, G.M. (1988) The relationship between gallbladder contraction and progesterone in patients with gallstones. *Am. J. Surg.* **155**, 147–51
113. Hould, F.S., Fried, G.M., Fazekas, A.G., Tremblay, S., Mersereau, W.A. (1988) Progesterone receptors regulate gallbladder motility. *J. Surg. Res.* **45**, 505–12
114. Ryan, J.P., Pellechia, D. (1982) Effect of progesterone pretreatment on guinea pigs gallbladder motility *in vitro*. *Gastroenterology* **83**, 81–5
115. Ryan, J.P., Pellechia, D. (1982) Effect of ovarian hormone pretreatment on guinea pigs gallbladder motility *in vitro*. *Life Sci.* **31**, 1445–9
116. Ryan, J.P., Pellechia, D. (1984) Effect of pregnancy on gallbladder contractility in guinea pigs. *Gastroenterology* **87**, 674–9
117. Behar, J., Kwang, Y., Thompson, W.R., Biancani, P. (1989) Gallbladder contraction in patients with pigment and cholesterol stones. *Gastroenterology* **97**, 1479–84
118. Shinitzky, M. (1984) Membrane fluidity and cellular functions. In: Shinitzky, M. ed. Physiology of membrane fluidity. pp. 1–51. Florida: CRC Press
119. Holzbach, R.T., Marsh, M., Tang, P. (1977) Cholesterolosis: physical-chemical characteristics of human and diet-induced canine lesions. *Exp. Mol. Pathol.* **27**, 324–38
120. Fisher, R.S., Rock, E., Malmud, L.S. (1985) Cholinergic effects on gallbladder emptying in humans. *Gastroenterology* **89**, 716–722
121. Nakano, J., McCloy, R.E. Jr, Gin, A.C., Nakano, S.K. (1975) Effect of prostaglandins E1, E2, F2alpha, and pentagastrin on the gallbladder pressure in dogs. *Eur. J. Pharmacol.* **30**, 107–12
122. Makata, K., Ashida, K., Nakayawa, K., Fujiware, M. (1981) Effects of indomethacin on prostaglandin synthesis and on contractile response of the guinea pig gallbladder. *Pharmacology* **23**, 95–101
123. Kotwal, C.A., Clanachan, A.S., Baer, H.P., Scott, G.W. (1984) Effects of prostaglandins on motility of gallbladders removed from patients with gallstones. *Arch. Surg.* **119**, 709–12
124. Chapman, W.C., Peterkin, G.A., LaMorte, W.W., Williams, L.F. (1989) Alterations in biliary motility correlate with increased gallbladder prostaglandin synthesis in early cholelithiasis in prairie dog. *Dig. Dis. Sci.* **34**, 1420–24
125. Fridhandler, T.M., Davison, J.S., Shaffer, E.A. (1983) Defective gallbladder contractility in the ground squirrel and prairie dog during the early stages of cholesterol gallstone formation. *Gastroenterology* **85**, 830–6

126. Li, Y.F., Moody, F.G., Weisbrodt, N.W., Zalewsky, C.A., Coelho, C.U., Senninger, N., Gouma, D. (1986) Gallbladder contractility and mucus secretion after cholesterol feeding in the prairie dog. *Surgery* **100**, 900–4
127. Pomeranz, I.S., Shaffer, E.A. (1985) Abnormal gallbladder emptying in a subgroup of patients with gallstones. *Gastroenterology* **88**, 787–91
128. Lee, S.P., Maher, K., Nichols, J.F. (1988) Origin and fate of biliary sludge. *Gastroenterology* **94**, 170–176
129. Tera, H. (1960) Stratification of human gallbladder bile *in vivo*. *Acta. Chir. Scand. Suppl.* **256**, 4–85

*(Accepted by S. Bengmark 9 May 1990)*

